# Single-trial neuromagnetic analysis reveals somatosensory dysfunction in chronic Minamata disease

**DOI:** 10.1016/j.nicl.2023.103422

**Published:** 2023-05-03

**Authors:** Masaaki Nakamura, Samu Taulu, Hisateru Tachimori, Yui Tomo, Takahiro Kawashima, Yoko Miura, Mina Itatani, Shozo Tobimatsu

**Affiliations:** a Department of Clinical Medicine, National Institute for Minamata Disease, Kumamoto, Japan; b Department of Physics, University of Washington, Seattle, WA, USA; c Institute for Learning and Brain Sciences, University of Washington, Seattle, WA, USA; d Department of Clinical Data Science, Clinical Research & Education Promotion Division, National Center of Neurology and Psychiatry, Tokyo, Japan; e Endowed Course for Health System Innovation, Keio University School of Medicine, Tokyo, Japan; f Department of Orthoptics, Faculty of Medical Science, Fukuoka International University of Health and Welfare, Fukuoka, Japan

**Keywords:** Central somatosensory disturbance, Somatosensory function, Single-trial analysis, Magnetoencephalography, Methylmercury poisoning, Minamata disease

## Abstract

Methylmercury pollution is a global problem, and Minamata disease (MD) is a stark reminder that exposure to methylmercury can cause irreversible neurological damage. A “glove and stocking type” sensory disturbance due to injured primary sensory cortex (SI) (central somatosensory disturbance) is the most common neurologic sign in MD. As this sign is also prevalent in those with polyneuropathy, we aimed to develop an objective assessment for detecting central somatosensory disturbances in cases of chronic MD.

We selected 289 healthy volunteers and 42 patients with MD. We recorded the sensory nerve action potentials (SNAPs) and somatosensory evoked magnetic fields (SEFs) to median nerve stimulation with magnetoencephalography. Single-trial epochs were classified into three categories (N20m, non-response, and P20m epochs) based on the cross-correlation between averaged sensor SEFs and individual epochs. We assessed SI responses (the appearance rate of P20m [P20m rate] and non-response epochs [non-response rate]) and early somatosensory cortical processing (N20m amplitude, reproducibility of N20m in single-trial responses [cross-correlation value], and induced gamma-band oscillations of the SI [gamma response] of single epochs excluding non-response epochs). Receiver operating characteristic curve analyses were used to examine the diagnostic accuracy of each parameter.

We found that SNAPs exerted a marginal effect on the N20m. The N20m amplitude, cross-correlation value, and gamma response were significantly reduced in the MD group on either side (p < 0.0001), suggestive of altered early somatosensory cortical processing. Interestingly, the P20m rate and non-response rate were significantly increased in the MD group on either side (p < 0.0001), thereby suggesting impaired SI responses. Notably, P20m and absent N20m peaks were observed in 6 and 11 patients with MD, respectively, which may be attributed to increased numbers of P20m epochs. The cross-correlation value exhibited the highest correlation with the P20m rate or non-response rate. Thus, reduced reproducibility of N20m may play an important role in chronic MD. The cross-correlation value exhibited the highest correlation with the gamma response for both SI parameters in early somatosensory cortical processing. The area under the curve was > 0.77 (range: 0.77–0.79) for all parameters. Their confidence intervals overlapped with each other; thus, each SEF parameter likely had an approximately equivalent discrimination ability.

In conclusion, chronic MD is characterized by impaired SI responses and alterations in early somatosensory cortical processing. Thus, single-trial neuromagnetic analysis of somatosensory function may be useful for detecting central somatosensory disturbance and elucidating the relevant pathophysiological mechanisms even in the context of chronic MD.

## Highlights

• Minamata disease (MD) is a neurological disease with sensory symptoms caused by methylmercury poisoning.
• We analyzed single-trial neuromagnetic somatosensory responses (N20m, non-response and P20m epochs).
• Rates of P20m and non-response epochs were significantly increased in chronic MD.
• P20m and absent N20m may depend on the numbers and amplitude of P20m epochs.
• Early somatosensory cortical processing was impaired even in chronic MD.

## Graphical abstract

**Figure:**
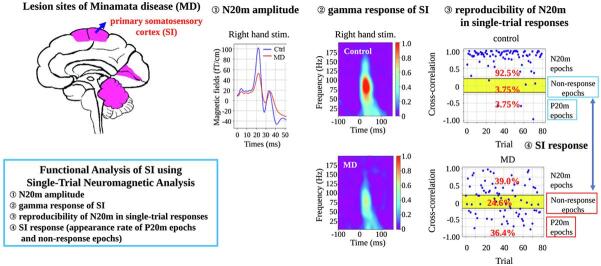

## 1 Introduction

Methylmercury (MeHg) is a major environmental neurotoxicant that damages the central nervous system depending on the dose and duration of exposure (Clarkson et al., 2003). In humans, accidental MeHg poisoning has been recorded in Japan (Tokuomi, 1961, Tokuomi et al., 1961, Maruyama et al., 2012) and Iraq (Bakir et al., 1973). In Japan, the industrial emission of MeHg resulted in MeHg poisoning in Minamata and Niigata, which was termed as Minamata disease (MD), first reported approximately 67 years ago.

MD is classified based on the onset time as acute/subacute MD (manifestation during the heavy contamination of seafood with MeHg in the Minamata Bay) and chronic MD (manifestation following the cessation of MeHg excretion). Acute or subacute MD is characterized by marked sensory disturbances in the distal parts of the extremities (“glove and stocking type” sensory disturbance), concentric constriction of visual fields, ataxia, and auditory disturbances. Except for sensory disturbance, neurologic signs are milder and less frequent in chronic MD than in acute or subacute MD (Uchino and Araki, 1984).

Given that chronic exposure mercury and its compounds can cause irreversible neurological damage and recent increases in environmental mercury emissions, the Minamata Convention on Mercury was held in August 2017, aiming to issue guidance on protecting human health and decreasing anthropogenic mercury emissions (The Lancet, 2017). Zhang et al. estimated the cumulative global cost associated with MeHg exposure during 2010–2050 as 19 trillion USD (Zhang et al., 2021) and suggested the need for preventive measures. An objective assessment of the neurologic signs in MD can provide insights into the pathophysiology of MeHg poisoning.

Clinically, sensory disturbance appears initially (Tokuomi, 1961, Tokuomi et al., 1961, Bakir et al., 1973, Maruyama et al., 2012) and has the lowest threshold in MeHg poisoning (threshold body burden of MeHg at which the symptoms appear: paresthesia, 25 mg; ataxia, 55 mg; dysarthria, 90 mg; and deafness, 170 mg) (Bakir et al., 1973). Neuropathologically, the postcentral gyrus (primary sensory cortex [SI]), precentral cortex, visual cortex, auditory cortex, and cerebellum are preferentially disturbed in acute or subacute MD (Takeuchi et al., 1962), whereas the lesions tend to localize in the SI and occipital lobe in chronic MD (Eto and Takeuchi, 1978). Thus, central somatosensory disturbance owing to injured SI forms the core of neurologic signs in MD.

As the “glove and stocking type” sensory disturbances frequently observed in MD (Uchino et al., 1995) are also prevalent in forms of polyneuropathy such as diabetes, a method for detecting central somatosensory disturbance is absolutely essential. In a study on somatosensory evoked potentials (SEPs), which scan the connectivity of peripheral sensory nerves to the SI, the N20 component, which arises from the SI, was absent in patients with acute or subacute MD (Tokuomi et al., 1982). However, another study demonstrated a normal N20 component in patients with chronic MD, despite somatosensory disturbances (Nagaki, 1984). These findings suggest that decreased amplitudes of N20 component in SEPs could only be recognized in patients with acute or subacute MD with stronger pathological findings than chronic MD. Since the participants of this study were patients with chronic MD, we considered that developing a more sensitive way of detecting central somatosensory disturbances was necessary. Therefore, we used magnetoencephalography (MEG) because of its excellent spatial resolution compared with SEPs resolution.

MEG can detect magnetic fields generated by neural currents with marginal distortion owing to differences in conductance at a millisecond temporal resolution (Hämäläinen et al., 1993). In somatosensory evoked magnetic fields (SEFs) to median nerve stimulation, the first signal (N20m; a counterpart of the N20 component of SEPs) peaks at approximately 20 ms over the contralateral SI, followed by deflections of opposite polarity at approximately 30 ms (P35m). Magnetic signals are less significantly affected by the presence of the brain, skull, and scalp than electric potential distributions. Further, the duration of stimulus artifacts for SEFs is shorter than that for SEPs (Kakigi and Forss, 2010). Consequently, MEG is a robust method for estimating single-trial somatosensory responses (Sander et al., 2005).

In this study, we aimed to develop a highly sensitive objective assessment for detecting central somatosensory disturbance in patients with chronic MD. We intended to perform single-trial analysis of SEFs using MEG to clarify the pathophysiological mechanisms underlying central somatosensory disturbances in chronic MD. Single-trial epochs were classified into three categories (N20m, non-response, and P20m epochs) based on the cross-correlation between average sensor SEFs and individual epochs. Further, we compared the characteristics of N20m between patients with MD and healthy controls given previous reports of stable N20m latency and amplitude in healthy participants (Ou et al., 2007). In terms of N20m characteristics, we examined the SI response (appearance rate of P20m [P20m rate] and non-response epochs [non-response rate]) and early somatosensory cortical processing (N20m amplitude, reproducibility of N20m at single-trial responses [cross-correlation value], and induced gamma-band oscillations of the SI [gamma response] of single epochs excluding non-response epochs). Our analyses revealed altered SI responses and somatosensory function in patients with chronic MD. These neuromagnetic parameters provide objective evidence for central somatosensory disturbances in chronic MD and may be helpful in elucidating the pathophysiological mechanisms of central somatosensory disturbance.

## 2 Materials and methods

### 2.1 Participants

**Fig. 1:**
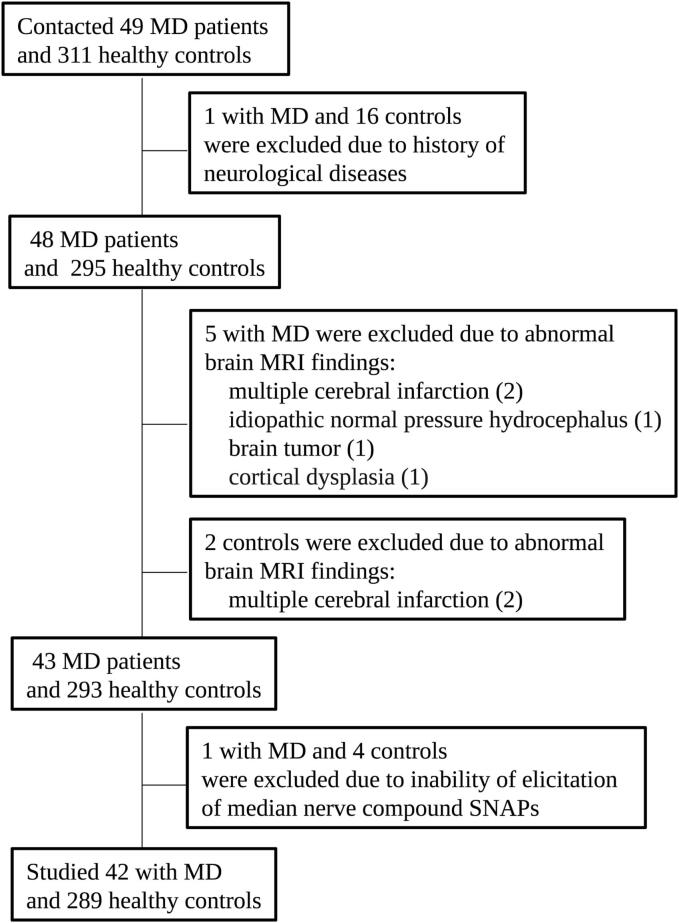
Flowchart of participant recruitment and enrollment. MD = Minamata disease.

Between April 2010 and October 2022, we recruited 42 patients with MD (20 women, 22 men; mean age 70.0 ± 10.6 years) and 289 healthy volunteers (131 women, 158 men; mean age 67.7 ± 9.4 years). Fig. 1 depicts the eligibility criteria and participant flow, including detailed reasons for exclusion. Healthy age- and sex-matched volunteers were recruited as controls from the non-MeHg-polluted areas (principally Kumamoto city). MD was officially confirmed according to the criteria of acquired MD and infant MD, including a history of exposure to MeHg from fish and shellfish consumption, and the presence of neurologic symptoms (sensory disturbances along with cerebellar ataxia, concentric constriction of visual fields, or otologic disturbances) (Director General of Environmental Health Department, 1986). Infant MD included both fetal MD due to placental MeHg exposure and postnatal MD owing to the oral ingestion of MeHg after birth. We included 15 patients with acquired MD and 27 with infant MD. The exclusion criteria were as follows: 1) a history of neurologic diseases; 2) evidence for organic changes accompanying neoplasms, demyelination, bleeding, and infarction on brain magnetic resonance imaging (MRI) scans; and 3) unsuccessful elicitation of median nerve compound sensory nerve action potentials (SNAPs).

### 2.2 MEG recording

The right and left median nerves were stimulated at the wrist in separate recordings using constant current pulses of 0.2-ms duration. Stimulus intensity determined by visual inspection was 1.5 times the motor threshold to evoke twitches of the thumb. The interstimulus interval was 3.7 s to avoid habituation to SII responses.

### 2.3 MR image acquisition

The three-dimensional (3D) T1-weighted images were acquired using a 1.5 T MRI unit (Magnetom Symphony, Siemens Healthcare, Erlangen, Germany) or a 3 T MRI unit (Discovery MR750 3.0 T, GE Healthcare, Milwaukee, WI).

### 2.4 Data acquisition

We recorded the SEF responses using a whole-head 306-channel sensor array (Vectorview, ELEKTA Neuromag, Helsinki) that comprises 204 planar gradiometers and 102 magnetometers. We analyzed the MEG data recorded by 204-channel planar-type gradiometers. Before the recording, three anatomical fiducial points (nasion and bilateral preauricular points) and four head position indicator coils on the scalp were digitized using a 3D digitizer. The sampling rate was set at 5,000 Hz, and the recording bandpass ranged from 0.03 to 1,500 Hz. We collected 80–91 responses per each median nerve stimulation, and 62–91 artifact-free trials were used for the analyses. Further, we recorded the orthodromic median nerve SNAPs from the surface electrodes placed on the elbow to estimate the contribution of peripheral sensory nerve impairment to the sensory disturbance in chronic MD.

### 2.5 Data processing

Because this study focused on the response of N20m, we applied a band pass filter (6–200 Hz) to the preprocessed data using MNE-Python (v0.20) (Gramfort et al., 2013).

We compared neural responses (P20m rate and non-response rate) and early somatosensory cortical processing (N20m amplitude, cross-correlation value, and gamma response) between patients with MD and controls. Using source waveforms obtained from equivalent current dipole modeling, we estimated the locations and activation strengths of the neural generators of N20m.

We performed the following three steps to estimate the SI function accurately: First, we needed to select the MEG sensor that best reflected the SI function. This is because data analysis is significantly affected by the choice of the sensor. We selected the sensor with the highest similarity of the averaged sensor SEFs to the source waveform reflecting neural activity in the SI, using the cross-correlation between the sensor SEFs and source waveform at N20 peak latency ± 3.0 ms. N20m amplitudes of the source waveform were highly correlated with those of the sensor SEFs (Fig. S1); therefore, the sensor SEFs were considered to reflect the source waveform. In case of an absent N20m peak, where the N20m dipole was estimated at a position widely different from the anatomical SI position owing to low amplitude, we selected sensor 1133 (right hemisphere) or 0443 (left hemisphere).

Second, we analyzed the SEFs under the restricted vicinity of the N20m peak (peak latency ± 3.0 ms). This is because N20m is a very stable response and is unaffected by the conscious state (Ou et al., 2007). Third, we denoised the raw data using a combination of two methods to increase the signal-to-noise ratio (SNR). Following denoising by the oversampled temporal projection (OTP) method, a recently developed method for the suppression of sensor noise and artifacts (Larson and Taulu, 2018), we performed the temporal signal space separation (tSSS) method, which removes artifact signals arising outside the sensor helmet, even if the artifact source is very close to the sensor array (Taulu and Simola, 2006). The combined use of OTP and tSSS is valuable for obtaining a higher SNR signal of a single-trial epoch and improves the detectability of high-frequency SEFs (Clarke et al., 2020).

**Fig. 2:**
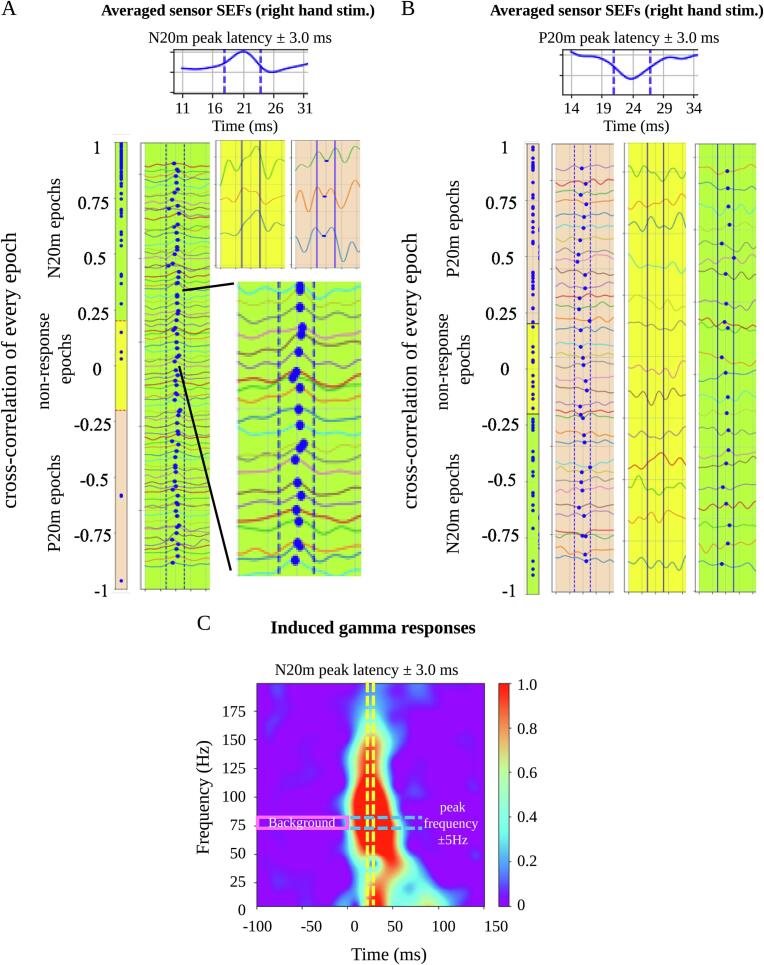
Analysis of SEFs. The cross-correlation was calculated between the averaged sensor SEFs and SEFs at single-trial epochs in the N20m peak latency ± 3.0 ms. Three categories of single-trial epochs based on the cross-correlation of individual epochs were determined as follows: N20m, P20m, and non-response epochs. For the N20m peak at SEFs, we defined a cross-correlation of > 0.2, −0.2 to 0.2, and <  − 0.2 as N20m (green zone), non-response (yellow zone), and P20m epochs (orange zone), respectively (**A**). For the P20m peak at SEFs, we defined cross-correlations of > 0.2, −0.2 to 0.2, and <  − 0.2 as P20m (orange zone), non-response (yellow zone), and N20m epochs (green zone), respectively (**B**). The mean cross-correlation of each single-trial epoch excluding non-response epochs was defined as the cross-correlation value. **(C)** Time-frequency analysis was performed in single-trial epochs, excluding non-response epochs. The power strength of the induced gamma oscillations was calculated from the average of 6 ms centered around the N20m latency and that of 10 Hz centered around the peak frequency value of gamma oscillations. The relative proportion of power strength divided by the baseline power strength (−100 to 0 ms) was log-transformed. SEFs = somatosensory evoked magnetic fields. (For interpretation of the references to colour in this figure legend, the reader is referred to the web version of this article.)

To quantify the reproducibility of N20m at single-trial responses in the selected sensor, we calculated the cross-correlation in the N20m peak latency ± 3.0 ms between the average sensor SEFs and SEFs at each single-trial epoch. Single-trial epochs were classified into three categories according to the cross-correlation of the individual epochs. For the N20m peak at SEFs, we defined cross-correlation of > 0.2, between − 0.2 and 0.2, and <  − 0.2 as N20m, non-response, and P20m epochs, respectively (Fig. 2A). For the P20m peak at SEFs, we defined cross-correlation of > 0.2, between − 0.2 and 0.2, and <  − 0.2 as P20m, non-response, and N20m epochs, respectively (Fig. 2B). The rate of the non-response epochs was evaluated as the non-response rate. The rate of the P20m epochs was evaluated as the P20m rate. The mean cross-correlation of every single-trial epoch without non-response epochs was defined as the cross-correlation value.

We estimated the N20m amplitude in the averaged sensor SEFs and gamma response at the single-trial epochs excluding non-response epochs. The positive value of the N20m amplitude was defined as the deflection of N20m from baseline. To assess the gamma response, each raw single-trial epoch was analyzed by the Morlet wavelet-based time–frequency analysis from 6 Hz to 200 Hz at 1-Hz steps (Fig. 2C) using MNE-Python (v0.20) (Gramfort et al., 2013). Peak frequency values of induced gamma oscillations were defined as frequency values that displayed the largest power strength within the time windows of N20m peak latency ± 3.0 ms. The power strength of induced gamma oscillations was calculated from the average of 6 ms centered around the N20m latency (3.0 ms before and 3.0 ms after N20m latency) and that of 10 Hz centered around the peak frequency value of gamma oscillations (5 Hz higher and 5 Hz lower than the peak frequency value). The relative proportion of power strength divided by baseline strength (−100 ms to 0 ms) was log-transformed.

### 2.6 Statistical analyses

For each MEG parameter, the non-parametric Mann–Whitney *U* test was used to compare data between patients with MD and controls. We used the Bonferroni correction for multiple pairwise comparisons of stimulation sides for each index. The result was considered statistically significant when the significance probability value was less than the adjusted significance level (0.05/2 = 0.025). We analyzed the correlations for each pair of SEF parameters on either side using Spearman’s correlation coefficients.

The receiver operating characteristic (ROC) curve describes a diagnostic trade-off between sensitivity and specificity with the adjustment of a discriminative threshold (Zou et al., 2007). The ideal diagnosis involves sensitivity, specificity, and area under the ROC curve (AUC) of 1 each, whereas an AUC of 0.5 indicates a chance level. AUC values of > 0.9, 0.7–0.9, and 0.5–0.7 represent high, moderate, and low diagnostic accuracy, respectively (Yang et al., 2018). Further, all features of the SEF parameters were integrated to one-dimensional features using linear discriminant analysis or logistic regression (LogReg). The 95% confidence intervals of the true positive rate were constructed using the bootstrap technique.

All tests were two-tailed, and statistical significance was set at a p-value of 0.05. All analyses, except ROC curve analysis, were performed using GraphPad Prism version 7.0 (GraphPad Software, San Diego, California, USA). ROC curve analysis was performed using Python 3.8.3 and scikit-learn 0.23.1, a Python machine learning library (Pedregosa et al., 2011).

### 2.7 Ethical declarations

This study was conducted in accordance with the ethical principles of the Declaration of Helsinki. This study was approved by the Ethics Committee of the National Institute for Minamata Disease (NIMD 10/001, 13/001, 15/001, 16/003, 22/002). Participation was voluntary. Written informed consent was obtained from all participants.

## 3 Results

### 3.1 Evaluation of peripheral sensory nerve function

The conduction velocity of the median nerve SNAPs was significantly slower in patients with MD than in controls on either side (left side: control: 53.7 ± 4.9 m/s; MD: 50.9 ± 5.0 m/s; p = 0.0001; right side: control: 53.6 ± 5.1 m/s; MD: 50.8 ± 5.1 m/s; p = 0.0002), despite adjustments for multiple comparisons via Bonferroni correction. There were no significant differences in the amplitude of the median nerve compound SNAPs between the controls and patients with MD on either side (left side: control: 21.5 ± 13.8 µV; MD: 23.5 ± 18.1 µV; p = 0.7886; right side: control: 17.8 ± 12.4 µV; MD: 18.4 ± 13.6 µV; p = 0.9517).

### 3.2 Neural responses and N20m amplitude

**Fig. 3:**
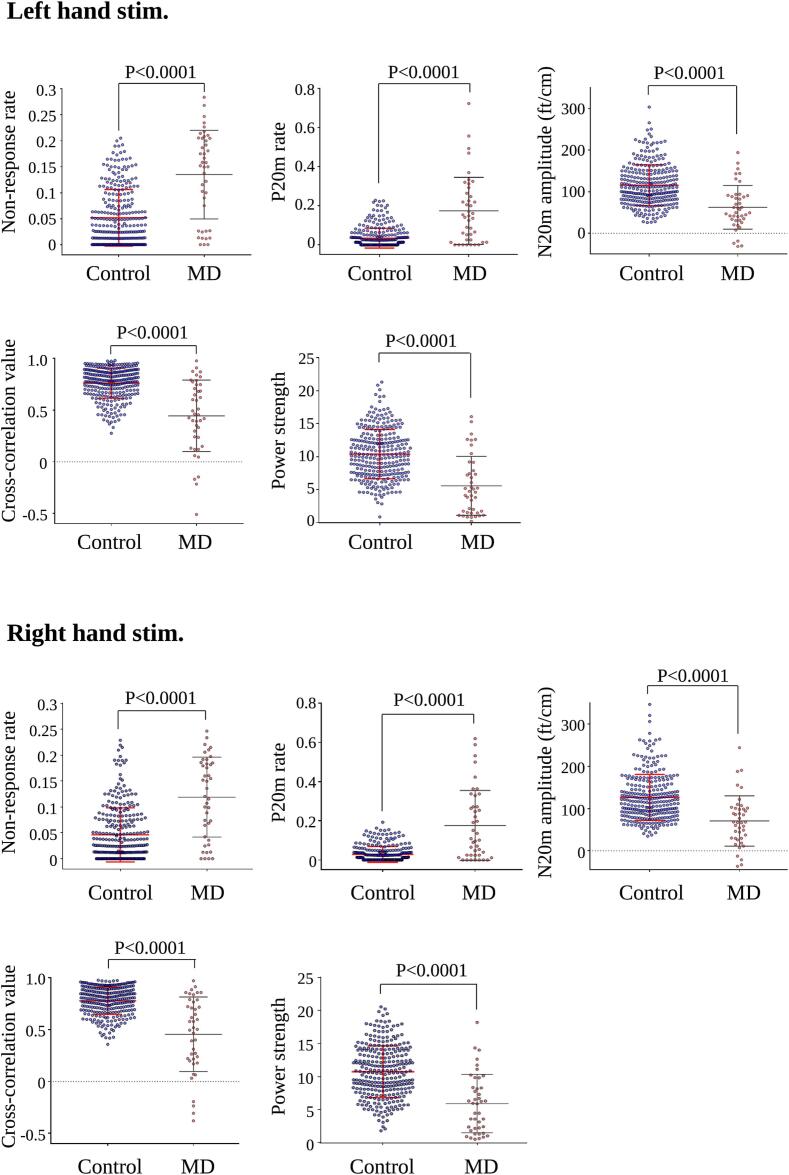
Comparisons of MEG findings between controls and patients with MD. Patients with MD demonstrate significantly higher non-response rates and P20m rates than controls on either side (p < 0.0001). The N20m amplitudes, cross-correlation value, and power strength of induced gamma oscillations were significantly lower in patients with MD than in controls on either side (p < 0.0001). Bars and error bars indicate mean and standard deviation, respectively. MEG = magnetoencephalography; MD = Minamata disease.

The P20m rate and non-response rate were significantly higher in patients with MD than in controls on either side, despite adjustments for multiple comparisons via Bonferroni correction (p < 0.0001) (Fig. 3).

**Fig. 4:**
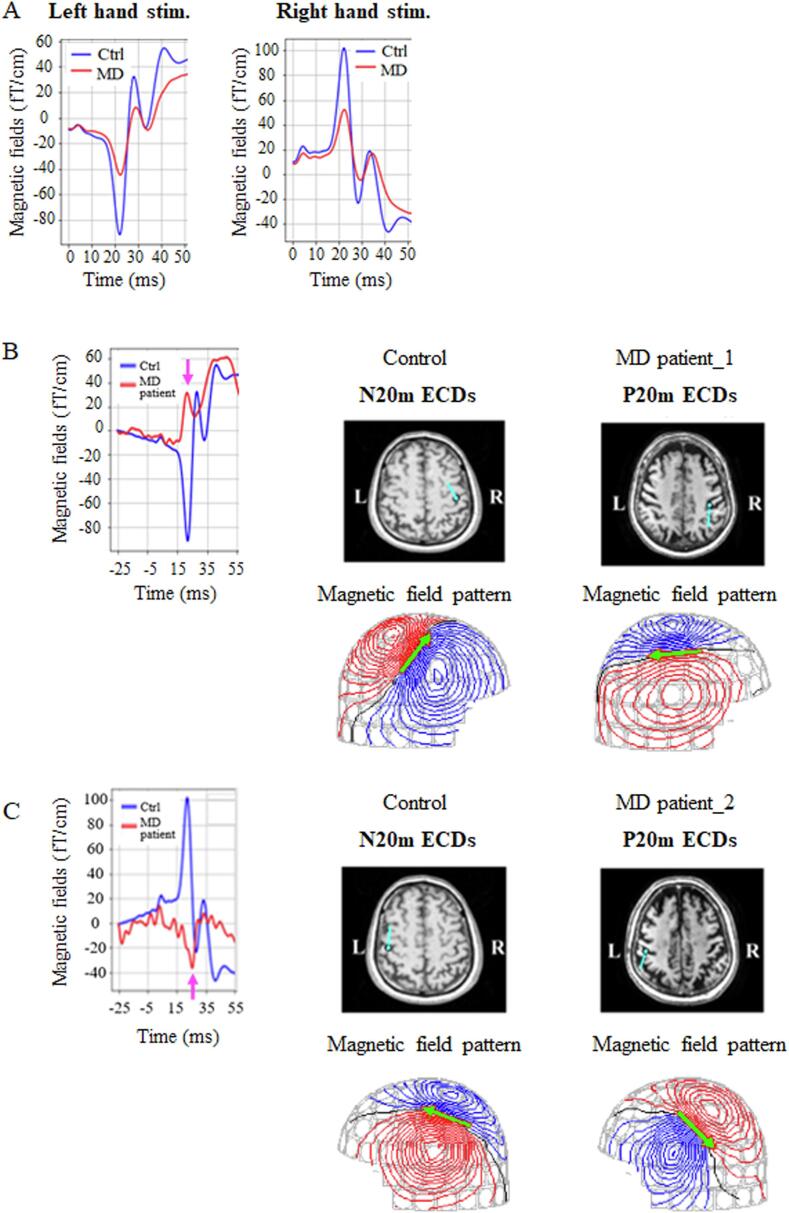
Amplitude and dipole direction of N20m. Compared with those in controls, the average N20m amplitudes in patients with MD were significantly decreased on either side **(A)**. The blue and red lines represent the average SEFs of controls and patients with MD, respectively. **(B)** and **(C)** present the examples of P20m in patients with MD. Pink arrows in the left diagram depict the P20m in patients with MD, whereas blue and red lines in the left diagram depict the averaged SEFs of controls and patients with MD, respectively. Blue lines on the brain MRI scans in the middle and right diagrams indicate the dipole direction of N20m in control and that of P20m in patients with MD, respectively. Green arrows on the magnetic field patterns represent the best-fitting ECDs in controls and patients with MD. SEFs = somatosensory evoked magnetic fields; ECDs = equivalent current dipoles; MD = Minamata disease; MRI = magnetic resonance imaging. (For interpretation of the references to colour in this figure legend, the reader is referred to the web version of this article.)

Compared with that in the control group, the average N20m amplitude in patients with MD was decreased on either side (Fig. 4A). We observed a significant reduction in the N20m amplitude in patients with MD on either side, despite adjustments for multiple comparisons with the Bonferroni correction (p < 0.0001) (Fig. 3). Notably, the N20m peak was absent in 11 patients with MD, and the P20m was observed in six patients with MD. Fig. 4B and C depict two typical examples of P20m.

### 3.3 Single-trial analysis of P20m and absent N20m peak

**Fig. 5:**
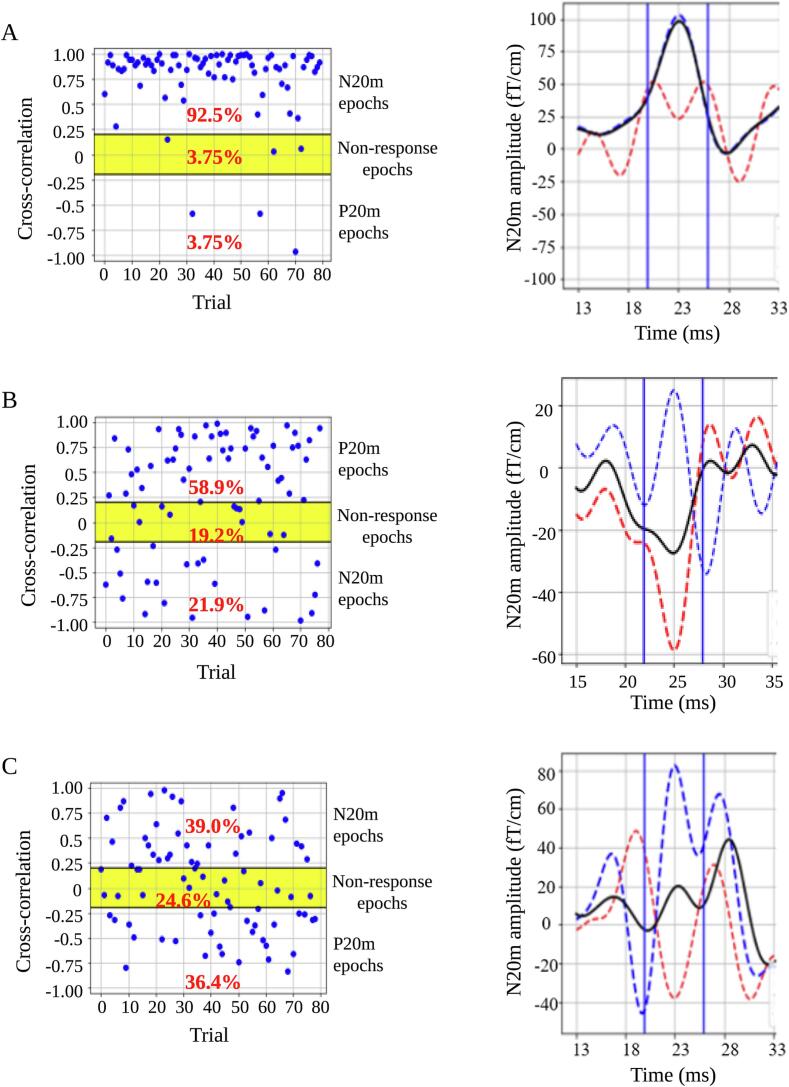
Single-trial analysis of P20m and absent N20m peak. Left diagrams depict the cross-correlation of each epoch. Right diagrams depict the averaged sensor SEFs (black line), averaged SEFs of N20m epochs (blue dashed line), and P20m epochs (red dashed line). The controls exhibited fewer P20m epochs (left diagram), and the averaged sensor SEFs and averaged SEFs of the N20m epochs almost overlapped (right diagram) **(A)**. In patients with MD exhibiting the P20m, there were more P20m epochs than N20m epochs (left diagram); the absolute value of the P20m amplitude of the averaged SEFs in the P20m epochs was higher than that of the N20m amplitude of the averaged SEFs in the N20m epochs (right diagram) **(B)**. In patients with MD exhibiting an absent N20m peak, the frequency of N20m and P20m epochs was similar (left diagram); the absolute value of the P20m amplitude of averaged SEFs in P20m epochs was lower than that of the N20m amplitude of averaged SEFs in N20m epochs (right diagram) **(C)**. SEFs = somatosensory evoked magnetic fields; MD = Minamata disease. (For interpretation of the references to colour in this figure legend, the reader is referred to the web version of this article.)

We examined the frequency of P20m epochs, N20m amplitude of averaged SEFs in N20m epochs, and P20m amplitude of averaged SEFs in P20m epochs in controls and patients with MD with the P20m or an absent N20m peak. The controls displayed fewer P20m epochs, and the averaged sensor SEFs and averaged SEFs of the N20m epochs nearly overlapped (Fig. 5A). The patients with MD exhibiting the P20m peak demonstrated a higher number of P20m epochs than of N20m epochs and a higher absolute value of the P20m amplitude of averaged SEFs in P20m epochs than that of the N20m amplitude of averaged SEFs in N20m epochs (Fig. 5B). The frequency of N20m and P20m epochs was similar in patients with MD exhibiting an absent N20m peak. The absolute value of the P20m amplitude of averaged SEFs in P20m epochs was lower than that of the N20m amplitude of averaged SEFs in N20m epochs (Fig. 5C).

### 3.4 Cross-correlation value of N20m

**Fig. 6:**
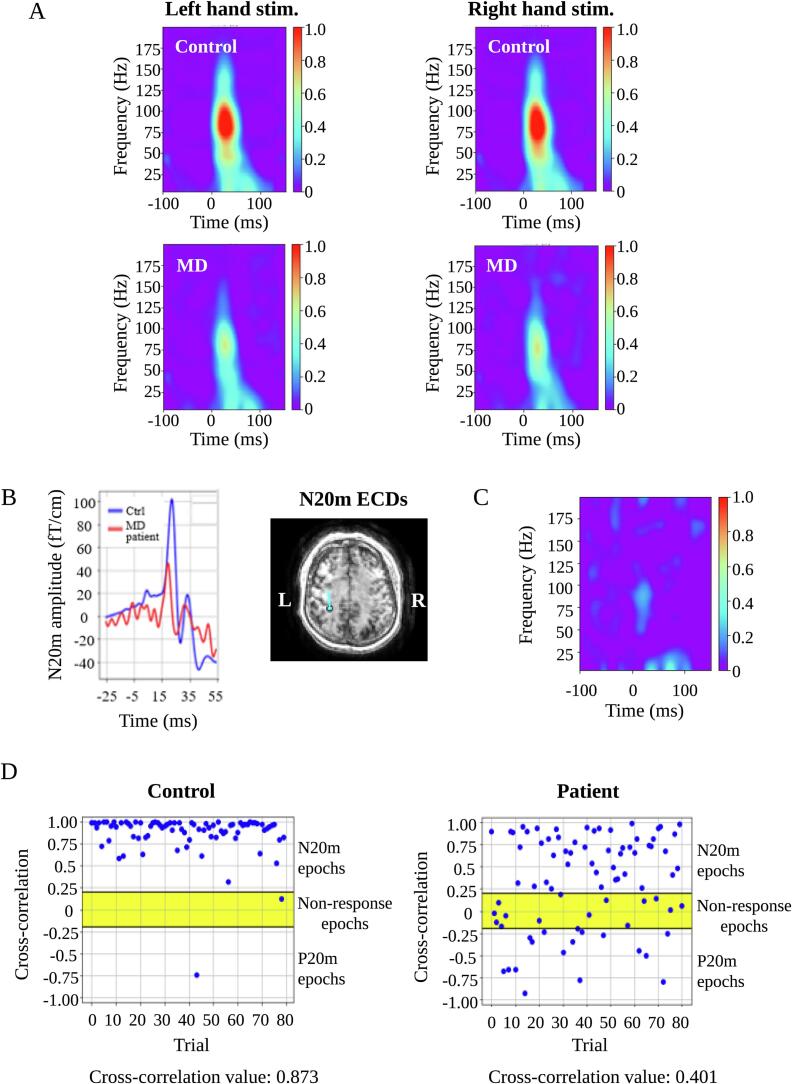
Time-frequency analysis of SEFs. Compared with that in controls, the power strength of induced gamma oscillations was significantly decreased on either side in patients with MD **(A)**. A representative case exhibiting the usefulness of analyzing the cross-correlation value of N20m and induced gamma oscillations. The N20m amplitude (left diagram) and location and direction of the N20m equivalent current dipole (right diagram) are normal **(B)**. However, reduced power strength of induced gamma oscillations **(C)**, cross-correlation values **(D)**, and increased number of non-response and P20m epochs **(D)** were observed. SEFs = somatosensory evoked magnetic fields; MD = Minamata disease.

We observed a significant reduction of the cross-correlation value in patients with MD on either side, despite adjustments for multiple comparisons via Bonferroni correction (p < 0.0001) (Fig. 3). Fig. 6 depicts a representative case in which the cross-correlation value was useful for detecting somatosensory dysfunction. In this case, the N20m amplitude was at the lower limit of the normal range values, with normal location and direction of the N20m equivalent current dipoles (Fig. 6B). Thus, the N20m was within normal limits using the conventional analysis. However, we observed a reduced cross-correlation value in this case (Fig. 6D). Similar to the findings shown in Fig. 6, we observed five cases with right-hand stimulation and eight with left-hand stimulation, whereas the opposite finding was found in two cases with right-hand stimulation and one with left-hand stimulation. Thus, the cross-correlation analysis was useful for detecting minute somatosensory dysfunction.

### 3.5 Time-frequency analysis of SEFs

Compared with that in controls, the power strength of induced gamma oscillations decreased on either side in patients with MD (Fig. 6A). The power strength of induced gamma oscillations was significantly lower in patients with MD than in controls on either side even after adjustment for multiple comparisons via Bonferroni correction (p < 0.0001) (Fig. 3). The power strength of induced gamma oscillations was markedly reduced in patients with MD (Fig. 6C) who displayed N20m within normal limits in the conventional analysis (Fig. 6B). Similar to the findings shown in Fig. 6, we noted two cases with right-hand stimulation and eight with left-hand stimulation, whereas the opposite finding was observed in only one case with left-hand stimulation. Thus, time–frequency analysis was useful for detecting minute somatosensory dysfunction.

### 3.6 Correlations between two SEF parameters

**Fig. 7:**
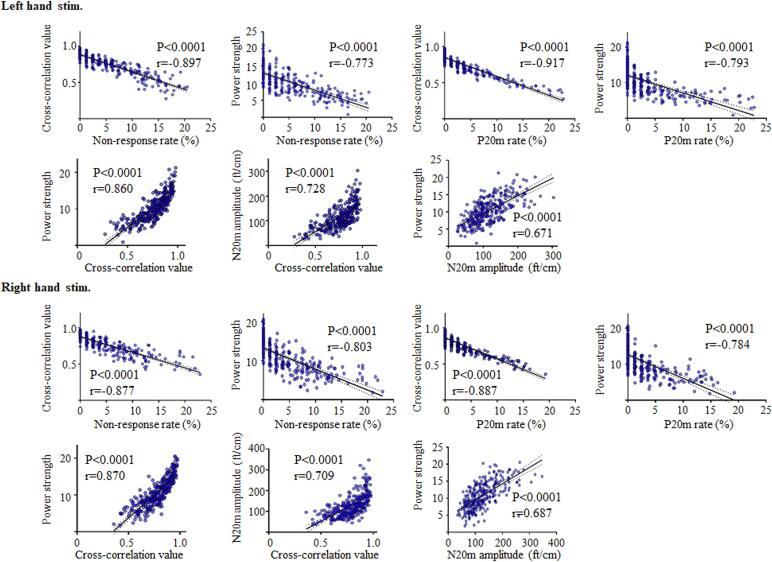
Correlations between two SEF parameters. On either side, the cross-correlation value exhibited the highest correlation with the non-response rate or P20m rate. High correlations were observed for both SEF parameters of early somatosensory cortical processing on either side. In particular, there was a strong correlation between the cross-correlation value and power strength of the induced gamma oscillations on either side. SEFs = somatosensory evoked magnetic fields.

The cross-correlation value and power strength of induced gamma oscillations on either side exhibited the highest negative correlation with the non-response rate and P20m rate (Fig. 7). The lower the cross-correlation value or the power strength of induced gamma oscillations, the greater the non-response rate and P20m rate. The correlation coefficient was > 0.671 (range; 0.671–0.870) for all SEF parameters in the early somatosensory cortical processing (Fig. 7), thereby suggesting a significant correlation between these parameters. In particular, we observed a strong correlation between the cross-correlation value and power strength of the induced gamma oscillations on either side.

### 3.7 Discrimination analysis

**Fig. 8:**
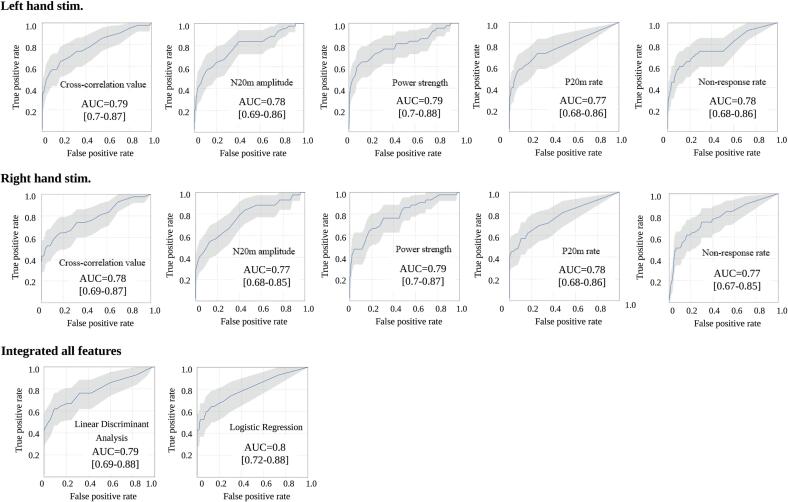
Receiver operating characteristic curves and corresponding area under the curve values for SEF parameters. The AUC was > 0.77 for all parameters, and the AUC value was the highest for the integrated SEF parameters using the LogReg method (0.8). Data are presented with 95% confidence intervals. AUC = area under the receiver operating characteristic curve; SEFs = somatosensory evoked magnetic fields; LogReg = logistic regression.

The AUC was > 0.77 (range; 0.77–0.79) for all parameters and was the highest (0.80 [range; 0.72–0.88]) for the integrated SEF parameters using the LogReg method (Fig. 8). The confidence intervals of the AUCs overlapped with each other; thus, every SEF parameter likely had an approximately equivalent discrimination ability.

## 4 Discussion

We performed single-trial analysis of SEFs to explore the pathophysiological mechanisms of central somatosensory disturbance in patients with chronic MD. Detailed analyses of SEFs under restricted vicinity of the N20m (peak latency ± 3.0 ms) characterized the altered SI response and early somatosensory cortical processing. In this study, the non-response rate and P20m rate were significantly higher in patients with MD than in controls, thereby indicating an impaired SI response. In addition, we observed a significant reduction of the N20m amplitude, cross-correlation value, and gamma response in patients with chronic MD, reflecting a functional abnormality of early somatosensory cortical processing. Therefore, these SEF parameters may shed light on the pathophysiology of minute central somatosensory disturbance in chronic MD.

Single-trial analyses have the potential to uncover meaningful brain dynamics that are obscured using the conventional method (averaging brain activity across trials) (Stokes and Spaak, 2016). However, compared with the conventional method, single-trial analyses have the disadvantage of low SNR. To overcome this drawback, researchers must obtain the high SNR waveform of a single-trial epoch. The combined use of OTP and tSSS enabled us to achieve this feature (Clarke et al., 2020). Eventually, we could dissect SI responses by classifying single-trial epochs (N20m, non-response, and P20m epochs). The non-response rate and P20m rate were significantly higher in patients with MD on both sides, suggesting an impaired SI response in chronic MD.

In this study, the N20m amplitude on either side was significantly reduced in patients with chronic MD (p *<* 0.0001). Further, we observed a reduction or disappearance of the N20m amplitude in patients with MD who displayed recovery of the sensory disturbance. Relatively slower peripheral conduction velocity of MD may affect the latency of N20m but not the N20m waveform and amplitude. We did not analyze N20m latency; therefore, peripheral SNAPs were not considered to exert a significant impact. N20m amplitudes are reduced in the acute phase of sensorimotor stroke (Wikström et al., 1999). However, a study has reported that reduced amplitudes do not recover despite regaining of the P35m amplitude during recovery from sensory disturbance in stroke (Wikström et al., 2000). In other words, the reduced N20m amplitude owing to the central somatosensory disturbance continued for an extended period. Therefore, it may be an indicator of the chronic phase of central somatosensory disturbance.

One may argue that it is unclear whether the strength of the correlation between the averaged sensor SEFs and SEFs at single-trial epochs results from the amplitude of SEFs or increase of the background 1/f noise in chronic MD. As shown in Fig. S2, the frequency component of ≤ 40 Hz becomes almost flat or slightly tilt in the 6-ms time window. Therefore, 1/f noise < 40 Hz appears not to contribute to such a short time scale peak. In addition, we only picked up good epochs with peaks in the short time of 6 ms, and cross-correlations were calculated from them in this study. Namely, epochs with no clear peak were excluded as bad epochs. Furthermore, differences in cross-correlation values by high pass filter frequency are small, 0.006 (Fig. S3). Taken together, the strength of the correlation between the averaged sensor SEFs and SEFs at single-trial epoch was less affected by background 1/f noise.

The P20m peak and an absent N20m peak were observed in 6 and 11 patients with MD, respectively. The latencies of N20m in the averaged SEFs of N20m and P20m epochs in the averaged SEFs of P20m epochs were almost identical (Fig. 5). Thus, SEFs of the N20m and P20m epochs counteracted the generation of the N20m peak. Patients with MD exhibiting P20m showed more P20m epochs than N20m epochs. The frequency of N20m and P20m epochs were similar in patients with MD exhibiting an absent N20m peak. Compared with the N20m amplitude of averaged SEFs in the N20m epochs, the absolute value of the P20m amplitude of averaged SEFs in P20m epochs was high in patients with MD exhibiting a P20m peak and low in those with MD exhibiting an absent N20m peak. Therefore, the difference between P20m and an absent N20m peak may depend on the numbers of P20m epochs and P20m amplitude of averaged SEFs in P20m epochs.

P20m is occasionally observed in unilateral polymicrogyria, a rare dysplastic disorder. Ishitobi et al. recognized P20m in two of five patients with unilateral polymicrogyria in the parietal region (Ishitobi et al., 2005). They speculated that P20m was generated by the unusual positive potential over the dysplastic cortex, despite an unknown mechanism. All six cases of P20m comprised patients with infant MD. Because the SEF response of school-age children and adolescents is closer to that of adults (Bast et al., 2007, Nevalainen et al., 2012; Nevalainen et al., 2014), P20m may appear during reorganization after the impairment of SI in the immature somatosensory system.

Researchers have recognized the decreased variability of neural activities across trials (increased reproducibility) as a general property of sensory perception (Churchland et al., 2010, Schurger et al., 2015). The cross-correlation value reflects the phase-shifting of the N20m peak among trials; therefore, it was considered to reflect neuronal synchronization in early somatosensory processing. This value was significantly reduced on both sides, thereby suggesting reduced neuronal synchronization during early somatosensory processing in chronic MD.

Gamma oscillations emerge from the precise synaptic interactions of excitatory pyramidal cells and inhibitory gamma-aminobutyric acid-ergic interneurons (Kann et al., 2014). They can be associated with the temporal binding of local and distant neuronal assemblies in the mammalian brain (Buzsáki and Draguhn, 2004: Womelsdorf et al., 2007). Induced oscillations are generated by some distinct high-order processes that are often described in terms of binding and/or neuronal synchronization (Singer and Gray, 1995, David et al., 2006). Hagiwara et al. reported on early gamma-band neuronal synchronization between the SI and secondary somatosensory cortex (SII) areas (Hagiwara et al., 2010). Thus, induced gamma oscillations reflect early neuronal synchronization between the SI and SII. The gamma response was significantly reduced on both sides in patients with chronic MD, suggesting impaired early neuronal synchronization between the SI and SII. Gamma-band synchronization has been considered to enhance the neuronal gain to relevant sensory input leading to more efficient downstream processing (van Es and Schoffelen, 2019). Thus, reduced gamma response may explain an abnormal two-point discrimination in approximately 70% of patients with chronic MD who present with intact superficial sensation of the upper extremities (Uchino et al., 2001).

The cross-correlation value and power strength of the induced gamma oscillations displayed the highest negative correlation with the non-response rate and P20m rate, suggesting that the reduced reproducibility of N20m and gamma oscillations played an important role in the impaired SI response in patients with chronic MD. For both SEF parameters involved in early somatosensory cortical processing, we observed a strong correlation between the cross-correlation value and power strength of the induced gamma oscillations. This finding may be attributed to their close neurophysiological actions (early neuronal synchronization in somatosensory processing).

We performed an ROC analysis to evaluate the ability of each parameter to discriminate between controls and patients with MD. The AUC was > 0.7 for all parameters; therefore, each SEF parameter displayed a moderate discrimination ability. Because the confidence intervals of the AUCs overlapped with each other, each SEF parameter was considered to exhibit an approximately equivalent discrimination ability. In other words, these SEF parameters may be useful for detecting chronic-phase central somatosensory disturbance.

Our study had some limitations. First, the relatively small sample size may reduce the statistical power of this study. The first patients with MD were officially identified in 1956, and the number of surviving patients is gradually decreasing (<225 survivors of confirmed Minamata disease in Kumamoto prefecture). We recruited 49 patients with MD; therefore, the current sample may be broadly representative of chronic MD. Second, we did not analyze other central somatosensory disorders, such as multiple sclerosis, stroke, and brain tumors. Organic changes in the somatosensory pathway running from the thalamus to the SI accompanied by neoplasms, demyelination, bleeding, and infarction are generally observed on brain MRI scans in patients with these diseases. In contrast, there are no organic changes other than brain atrophy in the SI in patients with MD (Korogi et al., 1998). Therefore, our MEG findings are likely useful for diagnosing chronic MD based on a combination of MRI findings, despite similar findings in central somatosensory disorders other than MD. Therefore, we aim to compare MEG or MRI findings between the two groups in future studies.

## 5 Conclusions

Functional abnormalities in early somatosensory cortical processing (reduced N20m amplitude, cross-correlation value, and SI gamma responses) as well as impaired SI responses (increased non-response rate and P20m rate) can be clearly detected in patients with chronic MD using MEG. Single-trial neuromagnetic analysis of somatosensory function may therefore be useful for identifying central somatosensory disturbance and elucidating the relevant pathophysiological mechanisms even in chronic cases of MD.

## Funding

This research did not receive any specific grant from funding agencies in the public, commercial, or not-for-profit sectors.

## CRediT authorship contribution statement

**Masaaki Nakamura:** Conceptualization, Data curation, Investigation, Project administration, Writing – original draft. **Samu Taulu:** Methodology, Supervision, Writing – review & editing. **Hisateru Tachimori:** Formal analysis, Writing – review & editing. **Yui Tomo:** Formal analysis, Writing – review & editing. **Takahiro Kawashima:** Formal analysis, Writing – review & editing. **Yoko Miura:** Investigation, Formal analysis. **Mina Itatani:** Investigation, Formal analysis. **Shozo Tobimatsu:** Methodology, Supervision, Writing – review & editing.

## Declaration of Competing Interest

The authors declare that they have no known competing financial interests or personal relationships that could have appeared to influence the work reported in this paper.

## Data Availability

The data that has been used is confidential.
