# Supplementary material for: Single-trial neuromagnetic analysis reveals somatosensory dysfunction in chronic Minamata disease
Source: Neuroimage Clin. 2023 May 3;38:103422. doi: 10.1016/j.nicl.2023.103422 (PMC10189551; doi:10.1016/j.nicl.2023.103422)
Supplement: Supplementary data 1 [file mmc1.docx]

**Supplementary Material**

1. **Correlation between the N20m amplifications of the source waveform and sensor SEFs**

N20m amplitudes of the source waveform were highly correlated with those of the sensor SEFs (Fig. S1); therefore, the sensor SEFs were considered to reflect the source waveform. We also performed single-trial analysis using sensor SEFs because single-trial analysis using source waveform was more complicated than the use of sensor SEFs.


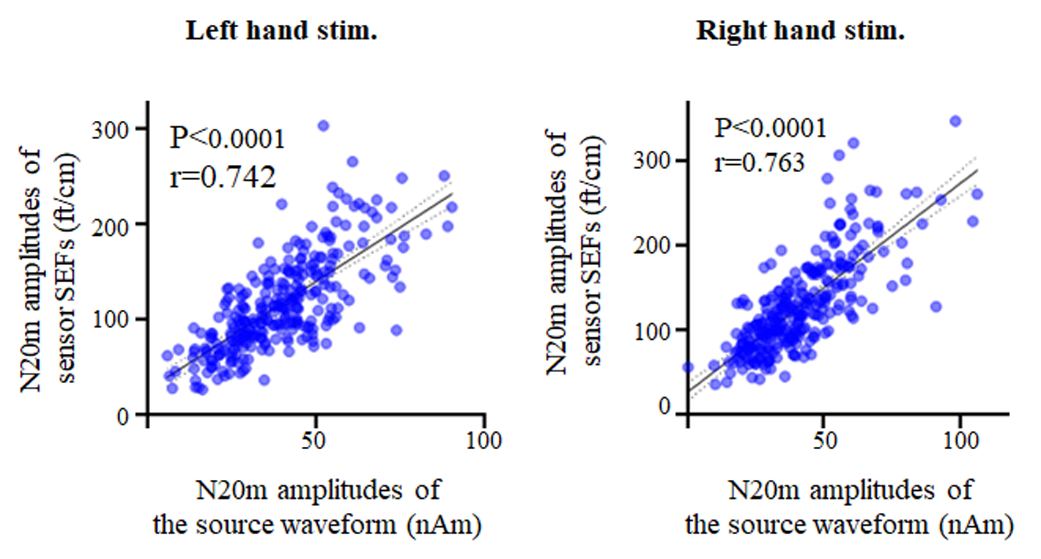


Fig. S1. Correlation between the N20m amplifications of the source waveform and sensor SEFs.

1. **Contribution of 1/f noise to single trial epoch**

As shown in Fig. S2, a frequency component of ≤ 40 Hz becomes almost flat or slightly tilted in the 6-ms time window. Therefore, 1/f noise < 40 Hz does not contribute to such a short time scale peak. Only epochs with peaks in the short time period of 6 ms were picked up as good epochs, and cross-correlations were calculated for them in this study. Note that epochs with no clear peak were excluded as bad epochs. As shown in Fig. S3, the differences in cross-correlation values by high pass filter frequency were small, 0.006. These findings suggested that the strength of the correlation between the averaged somatosensory evoked magnetic fields (SEFs) and SEFs at single-trials was less affected by background 1/f noise.


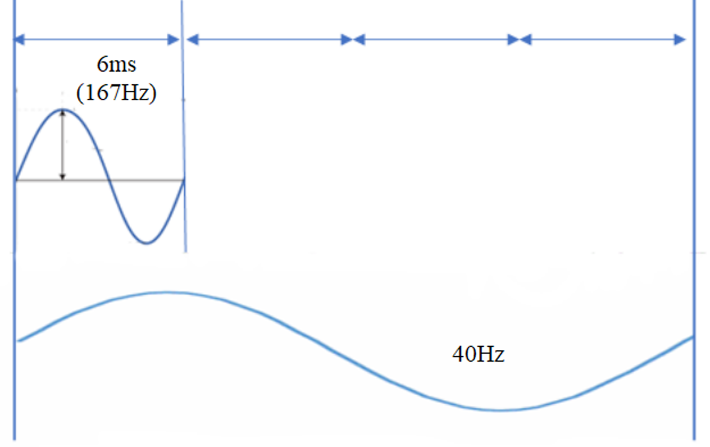


Fig. S2. Relationship between frequency and waveform.


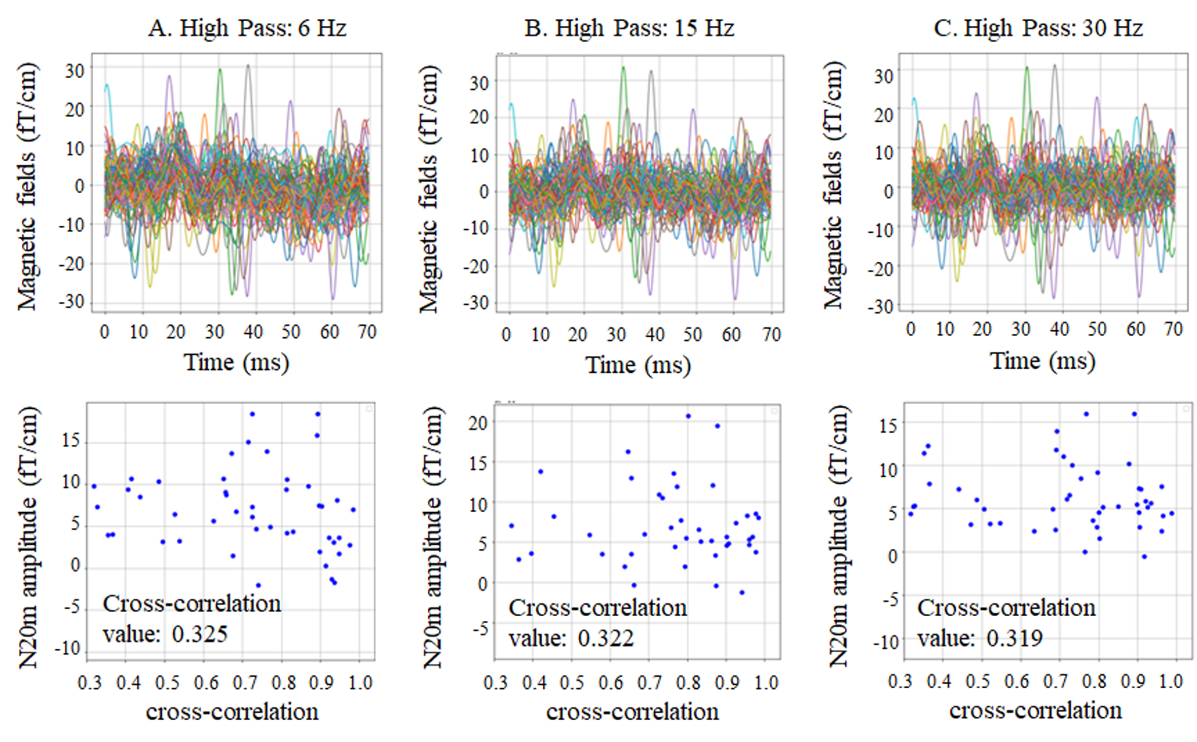


Fig. S3. Differences in cross-correlation values by high pass filter frequency.
